# Mitigating healthcare worker risk during the COVID-19 pandemic - experience from a large tertiary maternity centre in the United Arab Emirates

**DOI:** 10.25122/jml-2023-0168

**Published:** 2023-11

**Authors:** Chokkiyil Ponnambath Hafis Ibrahim, Ramza Al Chaer, Elsin Thomas, Stefan Weber

**Affiliations:** 1 Neonatal Division, Corniche Hospital, Abu Dhabi, United Arab Emirates; 2 Occupational Health Department, Corniche Hospital, Abu Dhabi, United Arab Emirates; 3 Union 71 Laboratory Services, Abu Dhabi, United Arab Emirates

**Keywords:** COVID-19, health care worker, mitigation, SARS-COV-2: Severe acute respiratory syndrome corona virus-2, COVID-19: Coronavirus disease 2019, WHO: World health organisation, UAE: United Arab Emirates, PPE: Personal Protective Equipment, HCW: Health care worker, PCR: Polymerase Chain Reaction

## Abstract

Since the beginning of the COVID-19 pandemic in 2020, healthcare workers (HCW) have been leading the charge in combating it, in spite of being disproportionately affected by the disease compared to the general population. This study describes the measures instituted at the largest maternity center in the United Arab Emirates during the pandemic to mitigate the risk of HCW contracting COVID-19, as well as the effectiveness of those measures. The incidence of COVID-19 amongst healthcare workers at the facility was compared to that in the general population over a 13-month period from February 2021 to February 2022. Data on population testing was obtained from the database maintained by the regional testing lab, while HCW testing data was obtained from the occupational health department at the hospital. The incidence of COVID-19 in HCWs and the general population was compared using summary statistics and charts. Several mitigation measures were instituted to protect HCW during the pandemic including patient traffic management, adequate provision of personal protective equipment, staff vaccination campaigns, infrastructure enhancement, workforce planning, and structured occupational health policies. During the study period the overall positivity rate in the general population was 5.78% (83,005/1.4 million tests) and that for staff was 1.19% (401/33,228 tests). The peaks and troughs on staff turning positive for COVID-19 coincided with peaks and troughs of the pandemic in the general population. The hospital instituted effective mitigation measures in protecting the staff and keeping COVID-19 rates well below the ones encountered in the general population.

## INTRODUCTION

Severe acute respiratory syndrome corona virus-2 (SARS-COV-2) is a novel virus which was first recognized in the Hubei province of China as causing a new corona virus disease in late 2019 [1]. The clinical syndrome caused by this virus has since been labelled Coronavirus disease 2019 (COVID-19). Since its discovery in Wuhan, the virus has spread rapidly across the world causing a global pandemic. The first confirmed case in United Arab Emirates (UAE) was reported in January 2020 [2]. The World Health Organisation declared it a global pandemic in March 2020 [3]. Internationally, healthcare workers have been disproportionately affected by the pandemic accounting for between 14-35% of infections while representing only 2-3% of the population [4].

Since the onset of the pandemic, the UAE government instituted a comprehensive package of measures to keep the population safe including early lockdowns, travel restrictions, mandatory mask wearing, restriction of gatherings, social distancing, robust contact tracing, and the scaling up and bolstering the healthcare infrastructure. In the healthcare sector several initiatives were mandated to protect staff and patients. These included designating healthcare facilities for COVID-19 care, temporary suspension of elective/non-emergency procedures, adequate provision of Personal Protective Equipment (PPE) for staff, periodic staff testing (the frequency of which changed based on community spread of COVID-19), and priority vaccination of healthcare staff and staff education [5].

Corniche Hospital is the UAE’s largest tertiary maternity hospital with an average of 6,000 deliveries per annum and around 1,000 admissions to the neonatal unit. In addition, the hospital provides comprehensive fertility and gynecological care. During the COVID-19 pandemic, the Corniche hospital became the designated facility in the public sector for managing pregnant women affected with COVID-19 within the Abu Dhabi region of the UAE. All pregnant women in the community who tested positive in the initial phases of the pandemic were admitted and monitored in the hospital or a quarantine facility until they tested negative. As the pressure on hospital and quarantine beds increased, a robust system of monitoring women at home with ready access to maternity care was instituted. In addition, all asymptomatic women who present to the hospital for care were screened for SARS-CoV2. From the start of the pandemic the hospital declared “code delta” i.e., declaration of a surge in infectious disease with serious adverse outcomes and started instituting a number of measures including the government mandated procedures. Throughout the pandemic, there has been an intense focus on staff safety.

In published literature, healthcare workers (HCWs) were found to be at a higher risk of infection compared to the general population. However, the reports are from parts of the world which were different in terms of resource allocation for healthcare infrastructure compared to the UAE. The aim of this research is to describe the staff safety measures instituted at Corniche hospital and assess the efficacy of these measures by comparing the number of staff who turned positive for COVID-19 to SARS-CoV2 PCR positivity in the community during the same period.

## MATERIAL AND METHODS

This was as a retrospective descriptive study, with the following primary objectives:

1. To describe the mitigation measures implemented at Corniche hospital to protect healthcare workers from COVID-19,
2. To compare the weekly incidence of COVID-19 in healthcare workers at Corniche Hospital to the general community,
3. To describe the total burden of COVID-19 disease in HCW at the hospital over the course study period.

The entire staff working at Corniche Hospital were eligible for inclusion. Data on staff testing was collated from the Corniche hospital occupational health department database. Community testing data was obtained from the testing database available at Union 71 laboratory. This is the public sector lab which processes majority of the COVID samples within the emirate and also had collated data on testing from up to 85% of other labs doing PCR testing within the Abu Dhabi region for reporting purposes. No patient identifiable data was collected or made available for the purpose of the research.

### Mitigation measures instituted at the hospital for staff safety

The measures instituted to reduce the risk of staff exposure is summarised in Table 1. Data comparison between the staff and general population (which included staff) datasets was done for a 13-month period from 13^th^ February 2021 to 15^th^ February 2022. During this period, there was regular staff testing, starting from February 2021, without any interruptions. During this time, there was a requirement for mandatory periodic testing for all residents in the emirate prior to accessing public and workspaces. If any of the staff members tested positive on tests conducted outside the hospital for any reason, they were added to both the numerator and denominator of the staff database. Staff vaccination data and infection data was collated from March 2020, when the pandemic was declared, up to February 2022.

**Table 1:** Salient mitigation measures instituted during the COVID-19 pandemic

**Patient and visitor traffic Management**
- Opening of Dedicated COVID-19 assessment centre for patients attending hospital for confirmed or suspected COVID-19 infection
- Designated inpatient ward for confirmed COVID-19 cases
- Universal screening for COVID of all patients being admitted to hospital and isolating pending results
- Exclusion zones while transporting COVID-19 patients within the hospital
- Exclusion of visitors during peaks of each pandemic wave (excluding exceptional circumstances for humanitarian reasons)
- Temperature checks on all staff and visitors to hospital
- Any visitor required to be COVID-19 negative as evidenced on their personalized ministry approved APP- Al Hosn which was the government mandated tracking and tracing app
**Infrastructure enhancement and environmental controls**
- Conversion of an entire ward into negative pressure rooms
- Conversion of operating rooms to negative pressure during surgery on COVID-19 patients
- Conversion of two designated rooms on labour ward to negative pressure for care of patients with COVID-19
- Portable high-efficiency particulate air (HEPA)-filters provided to all clinical areas
- Restriction of seat numbers in cafeteria with strict social distancing of two meters
- Removal of all automatic food dispensers
- Enhanced availability of hand gel around the hospital and enforcement by security at entrance
**PPE provision and use**
- Ensuring of adequate stocks of PPE for patients and staff across hospital with daily inventory monitoring and forward planning
- Universal masking introduced in hospital even before it was made a mandatory public health requirement
- Staff PPE use based risk of exposure to conserve essential stocks Those working in high-risk areas- emergency departments (ED),Labour and delivery suite( LDS), COVID-19 assessment centre and COVID ward- Full PPE including N95 masks and face shields Staff dealing with asymptomatic patients awaiting screen results- Mask and contact precautions Staff dealing with confirmed COVID-19 negative patients- Mask and universal precautions
**Workforce management**
- Leave suspended during peak of the pandemic waves and only essential travel abroad permitted to ensure adequate workforce
- Non-essential staff allowed to work from home
- Staff designated to work in COVID-19 areas with periodic rotation to prevent burnout
- All non-critical in person meetings cancelled
- Number of people in meeting rooms and offices restricted to ensure adequate social distancing
**Occupational health**
- Periodic screening of staff from weekly to fortnightly as recommended by state health regulator
- Strict isolation protocols for staff who tested positive or had close contact with positive cases with standardized return to work policies
- Priority staff vaccination with provision of vaccination clinic on-site
- Regular staff education and updates on safe practice
- Active contact tracing at work and monitoring if any staff member tested positive
**Leadership**
- Daily virtual leadership meetings chaired by CEO or proxy with key stake holders during the peaks of the pandemic to ensure standardised practice and adequate resource allocation

## RESULTS

**Figure 1:**
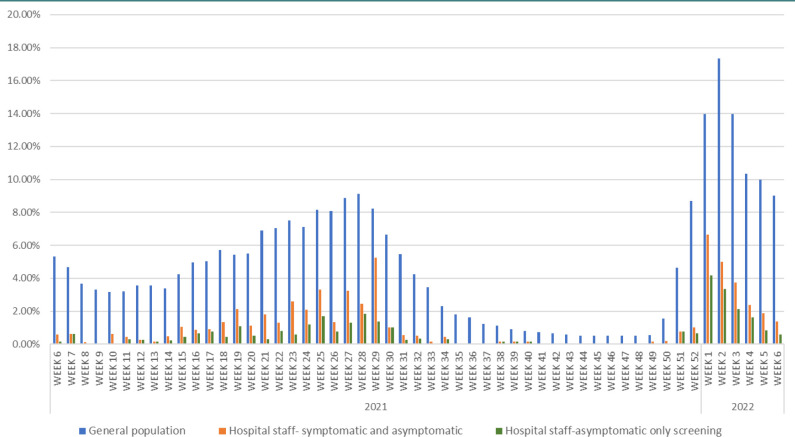
Weekly COVID PCR positivity rates- hospital staff v/s general population (Feb 2021-Feb 2022)

During the study period, the Union 71 database recorded 1.4 million PCR tests (including hospital staff and the general population) of which 83,005 were positive (5.78%). Amongst the staff at Corniche Hospital, 33,228 tests were performed on 1,129 people, of which 401 (1.19%) were positive. The comparison of weekly positive results for both populations is as displayed in Figure 1. As can be seen from the graph, the spikes of staff turning positive for COVID-19 coincided with the spikes of the pandemic in the general population. The proportion of staff who tested positive for COVID-19 was significantly lower compared to the general population for every week of the study period, with the rate being less than 50% of the community rates for the majority of the time. Over the course of the study period the positivity rate amongst staff members were only one fifth of that in the community (1.19% v/s 5.78%).

**Figure 2:**
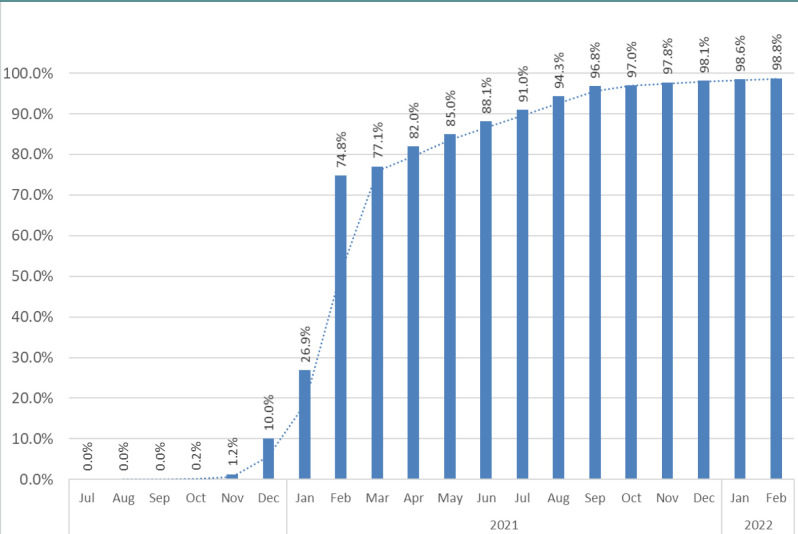
Proportion of hospital staff members completing at least 2 doses of SARS-CoV 2 vaccine

SARS-CoV2 vaccination was made available to the general population with healthcare professionals being categorized as a priority group when the Beijing Bio-Institute of Biological Products corona virus vaccine (BBIBP-CorV /Sinopharm^®^) vaccine became available in the country. The vaccine was approved by emergency authorization use by the ministry of health in September 2020 [6]. In the initial phases of the pandemic, the priority was to administer at least two doses of the vaccine to individuals to be considered fully vaccinated. As other vaccines became available (Pfizer Bio-Ntech-Comirnaty^®^, Gamaleya- Sputnik V^®^) they were added to the portfolio of available vaccines within UAE. All staff at our hospital received either the BBIBP-CorV or Pfizer Bio-Ntech-Comirnaty vaccine with onsite vaccination clinics being opened in November 2020. Staff also had access to all the other public health vaccination centers in the emirate. As can be seen in Figure 2 the vaccine uptake by the healthcare workers was high, with >80% of all staff having received at least 2 doses by April 2021.

Since the start of the pandemic, 495 (44%) of the staff had as SARS-CoV2 infection with doctors being the least affected group (Table 2). There were no deaths amongst staff members due to COVID-19 during the pandemic.

**Table 2:** Proportion of staff infected with SARS-CoV 2 from Mar 2020-Feb 2022

Job Categories	Total number of employees	Total staff tested COVID positive	Proportion
Medical	149	38	26%
Nursing	457	217	47%
Allied health	63	29	46%
Administrative	147	68	46%
External contractors	313	143	46%
Total	1,129	495	44%

## DISCUSSION

The COVID-19 pandemic has affected every section of society across the world causing significant morbidity, mortality, and suffering across populations. Healthcare workers have been at the forefront of fighting this pandemic and are one of the highest risk groups to contract the disease as part of their day-to-day work. They are also part of the community they live in. Public health and safety policies for containment of the pandemic would affect their risk of acquiring the disease like the rest of the population. Keeping HCWs safe has been a focus for governments across the world so that healthcare delivery is not compromised at a time of great need. This has been a prime focus of the government and the regulatory bodies in the emirate of Abu Dhabi as well. Many of the interventions to keep patients and healthcare workers at our facility safe during the pandemic were mandated by the government and local adaptations were made for optimum resource utilization.

The analysis of data from our hospital has shown that with all the interventions in place (in the community and hospital), we have been able to keep the rate of COVID-19 in our staff at a much lower level than in the general community. Though 44% of our staff got infected over the course of the pandemic (from March 2020- February 2022), this was staggered and the severity of illness in the vast majority of them was mild to moderate with no deaths. This has been vital in the continuation of services to our patients during the entire period.

The data from our center is different to what has been published from some of the other centers across the globe with regards to infection rates in HCWs. UAE is one of the few countries which instituted mass testing at scale across the population from quite early on in the pandemic. Moreover, the nation showcased with the highest testing rates per 1,000 inhabitants including healthcare workers in whom periodic surveillance was mandatory [2].

Most of the other countries instituted testing only for symptomatic cases and contact tracing during most of the pandemic with some centers implementing surveillance in HCWs later. Hence, in comparison to majority of published reports, the detection rate of asymptomatic infections in UAE in general is likely to be much higher- both in the general population and HCWs.

Rivet *et al*. from a large tertiary hospital in Cambridge, UK, reported a 3% COVID-19 RT-PCR positivity rate amongst 1,032 HCWs over a 3-month period at the beginning of the pandemic in 2020 [7]. The positivity rates in our cohort of HCWs was far lower during the entire course of the study period except for two weeks when it was higher than 3%. It is also notable that our study was conducted when the much more contagious Delta (B.1.617.2) and Omicron (B.1.1.529) variants were in circulation in the country. Marchese *et al*. reported the results of testing HCWs who were either symptomatic or had contact with a confirmed case of COVID-19 (3,572/9,265- 38% of all staff), from a large hospital in Brescia, Italy, over a 3-month period [8]. In that cohort infection was confirmed in 5.9% of all samples tested. If the testing was performed in all staff at the time, the positivity rates are likely to have been higher. The pattern was similar in a number of studies published from across the globe with point prevalence being much higher than in our study [9-11]. A meta-analysis of 39 studies on mass screening of asymptomatic healthcare workers over periods varying from one day to two months showed an average prevalence rate of 1.9% with a range of 0 -14.3% [12]. The point prevalence rates in our study were lower compared to majority of the studies included in the analysis.

From the data, we have shown that with a comprehensive package of measures we were to keep the point prevalence of COVID-19 amongst our HCWs to a much lower level than the general population. To our knowledge this is the first study to compare point prevalence rates of COVID-19 to the general population based on mass testing with SARS-CoV-19 RT-PCR. Previous reports of comparing incidence of COVID-19 in HCWs to that of the general population have used different methodologies. An app-based self-reported survey of the general population in the USA and UK reported a ten-fold higher incidence of COVID-19 amongst HCWs compared to the general population (3.9% v/s 0.33%) [13]. A survey based on Facebook^®^ users in the USA, on the other hand, revealed a slightly lesser risk of COVID-19 in HCWs compared to the general population, with a relative risk of 0.73 [14]. A cross sectional study based on seroprevalence in the city of New York early in the pandemic, found a lower prevalence amongst healthcare workers (9.8% v/s 16.7%) [15]. Even in comparison to studies quoted above the risk in our cohort of HCWs seem far lower at all time points during the study period, though the study methodology was different.

The reason for the rate of infections in our staff being lower than in the general population could be multifactorial. We had a strict policy of staff screening, contact tracing, and isolation procedures in place within a short time of the pandemic being declared. Healthcare care facilities with similar policies in other parts of the world have also reported low infection rates amongst their staff members [16]. In Abu Dhabi healthcare workers were considered a priority group to receive vaccination and multiple measures were instituted, including running on-site vaccination centers at healthcare facilities for staff members. At the start of the study period, 75% (Figure 2) of all our staff had already received at least two doses of the vaccine. This is much higher in comparison to general population in the emirate, which stood at 23% as per publicly available data [17]. We also hypothesize that HCWs by nature of their profession are likely to be more knowledgeable about the modes of transmission of the virus and hence adhere to prevention methods better than the general population.

## CONCLUSION

The pandemic has been a trying time for populations across the globe. Healthcare workers have been on the frontline for tackling this crisis. Like the rest of the world protecting and preserving the healthcare workforce has been a priority in the UAE. Our data shows that with a raft of prevention measures, the risk to healthcare workers can be reduced. The measures taken during this pandemic could act as a blueprint for any future pandemic with appropriate adaptations. To our knowledge this is one of the first studies reporting prevalence of COVID-19 in HCWs compared to the general population based on mass RT-PCR screening.

## Data Availability

Further data is available from the corresponding author on reasonable request.

## Personal Thanks

We would like to extend our gratitude and acknowledgement to infection prevention, education, facilities, and leadership teams at the hospital for adopting, implementing, and educating staff on the multiple interventions designed to reduce the risk of COVID-19 to healthcare workers in our hospital. We are grateful to Dr Paul Martin Bosio, chief medical officer from the Corniche Hospital, for reviewing the manuscript.
